# Minimally Invasive Minor Salivary Gland Biopsy for the Diagnosis of Amyloidosis in a Rheumatology Clinic

**DOI:** 10.1155/2014/354648

**Published:** 2014-02-23

**Authors:** Ridvan Mercan, Berivan Bıtık, Mehmet Engin Tezcan, Arif Kaya, Abdurrahman Tufan, Mehmet Akif Özturk, Seminur Haznedaroglu, Berna Goker

**Affiliations:** ^1^Division of Rheumatology, Department of Internal Medicine, Faculty of Medicine, Gazi University, Ic Hastalıklari ABD, Romatoloji BD, Besevler, 06500 Ankara, Turkey; ^2^Department of Rheumatology, Kartal Research and Training Hospital, 34890 Istanbul, Turkey; ^3^Department of Rheumatology, State Hospital, 20125 Denizli, Turkey

## Abstract

*Background*. Systemic amyloidosis is a potentially fatal condition, unless diagnosed and treated before development of irreversible organ damage. Demonstration of amyloid deposits within tissue biopsies is only definitive diagnostic method, which makes appropriate selection of biopsy site essential. Herein, we evaluated efficacy of minimally invasive minor salivary gland biopsy (MSGB) for the diagnosis of amyloidosis. *Methods*. We analyzed 37 biopsies taken from 35 patients. Suggestive findings for amyloidosis were significant proteinuria, renal impairment, refractory diarrhea, neuropathy, and restrictive cardiomyopathy. Minor salivary gland was the initial biopsy site in all subjects. When MSGB was negative but there was a high suspicion for amyloidosis, a kidney, duodenum, or rectal biopsy was performed for further investigation. *Results*. Mean age of patients was 45.4 and 21 were female. In 11 patients amyloidosis was diagnosed with MSGB. In overall 18 patients were diagnosed with amyloidosis. Sixteen of them were identified as being of AA type and two were AL type amyloidosis. The sensitivity of minimally invasive MSGB is 61.1% for diagnosing amyloidosis in this study. *Conclusion*. MSGB is a safe and simple method for the diagnosis of amyloidosis which can be performed in an outpatient setting. We suggest extensive use of this minimally invasive method.

## 1. Introduction

Amyloidosis is a potentially fatal condition characterized by extracellular deposition of nonbranching protein fibrils in organs [1]. This devastating condition is mainly caused by plasma cell disorders and numerous inflammatory diseases including autoimmune and chronic infectious diseases [2, 3]. Demonstration of amyloid deposits in biopsy specimens is the only way of establishing the diagnosis of amyloidosis [4, 5]. Therefore, appropriate selection of biopsy site is essential.

The sensitivity and specificity of histopathology varies greatly according to where the tissue biopsy is obtained [5, 6]. Sensitivity of biopsy samples from visceral organs is higher; however, it requires more invasive procedures with bearing higher risk of complications, such as bleeding, hematoma, and perforation. Abdominal fat pad, gingiva, and rectum are the most common initial biopsy sites because of their ease of accessibility, low complication rate, and lower costs [5]. However, diagnostic yield of these biopsies is somewhat lower compared to visceral organ biopsies.

Minor salivary glands have parenchymal and secretory components with considerable blood supply. Therefore, labial salivary glands are a good biopsy site for the demonstration of amyloid deposits [7]. Minimally invasive minor salivary gland biopsy (MSGB) is an easy procedure which can be performed by nonsurgical physicians with lower risk complications [5]. Since rheumatic diseases are a common cause of secondary amyloidosis and abovementioned advantages increased use of MSGB in rheumatology departments [8]. However, there is inconsistency in the literature about its utility and diagnostic yield [7, 9–11]. Hence, we aimed to investigate efficacy of this procedure for the assessment of amyloidosis in our patient population.

## 2. Methods

### 2.1. Patients

We have examined minimally invasive MSGB from 35 patients during the time period between January 2010 and September 2012 in this retrospective cohort study. All patients were recruited from the same rheumatology department and all had chronic rheumatic diseases. Demographic data, disease characteristics, comorbid conditions, and received treatments were recorded. All patients underwent a workup including renal and hepatic function tests, complete blood count, erythrocyte sedimentation rate, C-reactive protein, urinary protein excretion, and urinary cast examination.

The clinical or laboratory symptoms/findings suggestive of amyloidosis were presence of significant or worsening proteinuria, renal impairment, refractory diarrhea, neuropathy, and restrictive cardiomyopathy. Minor salivary gland was the initial biopsy site in all subjects. When MSGB was negative but there was a high suspicion for amyloidosis a kidney, duodenum, or rectal biopsy was performed for further investigation. Furthermore, other allied departments in the management of patients performed other tissue biopsies with their own decisions (i.e., duodenal/intestinal biopsy for diarrhea in gastroenterology or kidney biopsy for impaired renal function in nephrology). Disease course was followed for at least one year after the MSGB.

### 2.2. Biopsy Procedure and Histopathologic Study

All biopsies were carried out with minimally invasive technique, a modified technique defined by Friedman et al. [12]. In our method minor salivary glands of lower lip are palpated and local anesthesia (prilocaine) is applied with 30 gauge syringe. A 1-2 mm incision is made on lip mucosa near glandular salivary tissues. Salivary glands are extruded with opposite side pressure application. Procedure is completed with excision of salivary gland tissue with a scalpel. Homeostasis after incision was provided simply with 15 minutes of external compression without a suture.

Minor salivary glands were fixed in formalin, processed, embedded in paraffin, stained with Congo red, and examined by polarized light microscopy. Immunohistochemical examination was performed whenever indicated to discriminate AA and AL type amyloidosis. Pathologist was informed about presumed diagnosis of amyloidosis to improve diagnostic yield of biopsy.

## 3. Results

In defined time period 37 biopsies were performed from 35 patients with suspected diagnosis of amyloidosis. In 2 patients a rebiopsy was carried out 6 months after the initial biopsy. Mean age of patients was 45.4 and 21 were female (Table 1). The most common underlying disease was familial Mediterranean fever followed by ankylosing spondylitis and rheumatoid arthritis. The procedure was well tolerated in all patients without any major complication.

In 11 patients amyloidosis was diagnosed with MSGB (Table 2). Of these 30 patients had biopsy from other sites: duodenum, bone marrow, rectum. or kidney. Finally, overall 18 patients were diagnosed with amyloidosis in followup. Sixteen of them were identified as being of AA type and two were AL type amyloidosis.

## 4. Discussion 

Systemic amyloidosis is a progressive and fatal disease unless diagnosed early and treated if possible. Therefore, identification of disorder before occurrence of major clinical manifestations is imperative [13]. Demonstration of amyloid accumulation within tissues is the only means of diagnosis of amyloidosis, making tissue biopsy essential.

In our cohort we found sensitivity of MSGB as 61.1%. In systemic amyloidosis deposits can be found in any organ. Hence, tissue biopsies can be obtained from any organ but amyloid deposits range from massive to subtle in these organs [14]. Urinary protein excretion, an early finding of amyloidosis, was regularly ordered in our department for screening of proteinuria which was the most common indication of MSGB in our study. Moreover, deposits may not be present in tissue biopsies from compromised organs like kidney and salivary glands [15]. All these may explain low sensitivity of MSGB found in our study compared to other studies that report 83 to 100% sensitivity [2, 9, 16].

Since minimally invasive MSGB method is well tolerated it can be repeated sometime later after the initial procedure. We repeated MSGB 6 months after the first biopsy in two patients which confirmed the diagnosis of amyloidosis in one of them.

The major drawback of minimally invasive MSGB is that the amount of tissue obtained is relatively small [8, 12]. In our cohort the amount of salivary tissue was adequate for the histopathologic examination in all patients without major complications. The advantages of minimally invasive MSGB method are avoidance of more invasive methods, need for a small incision, any prerequisite for the procedure, quite low risk of bleeding and nerve damage, applicability in an outpatient setting, and rapid healing [1, 12]. Local and transient swelling, numbness, and pain were the only reported untoward effects by the patients related to the procedure.

We have a number of limitations in our study. A substantial number of our patients had FMF as underlying disorder which is somewhat different from other geographic regions [10, 16]. However, we do not think that it is a major concern since the mechanism leading to amyloidosis is persistent inflammation as observed in other rheumatic diseases [17]. Tissue biopsies other than MSGB were not routinely performed in all patients; therefore, we could not compare sensitivity and specificity of MSGB with other biopsy sites.

## 5. Conclusion

MSGB is a safe and simple method for the evaluation of amyloidosis which can be performed in an outpatient setting without preparation for procedure. We suggest extensive use of this minimally invasive method with consideration of its own drawbacks such as dependence of pathologists' skill and patients' amyloid load.

## Figures and Tables

**Table 1:** Demographic and disease characteristics of patients.

Characteristics	
Age, mean (SD)	45.4 (14.9)

Gender	21 female, 14 male

Main indication	
(i) Proteinuria	27
(ii) Impaired renal function	3
(iii) Refractory diarrhea	2
(iv) Cardiomyopathy	2
(v) Neuropathy	1

Underlying disorder	
(i) Familial Mediterranean fever	16
(ii) Rheumatoid arthritis	4
(iii) Ankylosing spondylitis	5
(iv) Collagenous tissue disorders	3
(v) Other	7

Other: Sjogren's syndrome 2, juvenile chronic arthritis 2, POEMS syndrome 1, rheumatic manifestation of plasma cell disorder 1, or enteropathic arthritis 1.

**Table 2:** Results of minor salivary gland biopsy.

MSGB	Confirmed amyloidosis	
	Amyloidosis (+)	Amyloidosis (−)
Amyloidosis (+)	11	0
Amyloidosis (−)	7	17

MSBG: minor salivary gland biopsy.
